# Male primary mediastinal choriocarcinoma with diffuse metastases: A case report

**DOI:** 10.1097/MD.0000000000016411

**Published:** 2019-07-12

**Authors:** Zhenhua Qiu, Yuanqiang Wu, Yapeng Wang, Chunhong Hu

**Affiliations:** Department of Oncology, The Second Xiangya Hospital, Central South University, Changsha, Hunan, China.

**Keywords:** diffuse metastases, male choriocarcinoma, primary mediastinal choriocarcinoma

## Abstract

**Rationale:**

Choriocarcinoma is a rare and highly invasive gestational trophoblastic tumor that secretes high levels of human chorionic gonadotropin (hCG). As one of the uncommon non-gestational choriocarcinoma, primary mediastinal choriocarcinoma is an exceeding rare, and aggressive malignancy with poor prognosis.

**Patient concerns:**

A 26-year-old man was admitted to the hospital with cough, shortness of breath, and occasional hemoptysis.

**Diagnoses and intervention:**

Imaging examinations revealed a large mediastinal mass, diffuse nodular opacities with blurred edges in both lungs, and multiple brain lesions. Laboratory tests showed an astonishing increase of serum β-hCG. A diagnosis of primary mediastinal choriocarcinoma with advanced lung and brain metastases was finally made after 3 biopsies and immunohistochemical analyses. Surgery and radiotherapy were not applicable at the time of diagnosis, and both targeted therapy and immunotherapy were unavailable. During the first 4 cycles of trophoblastic tumor-based chemotherapy, the patient improved clinically with fewer symptoms, decreased β-hCG and reduced lesions. However, drug resistance quickly emerged, forcing an alternative chemotherapy regimen that also failed.

**Outcomes:**

The patient finally endured symptoms including headache, dizziness and vomiting, and subsequently succumbed after an overall survival time of six and half months.

**Lessons:**

Male primary choriocarcinoma is an extremely rare type of malignancy. Greater awareness, earlier diagnosis and novel treatments are urgently needed to benefit patients.

## 1 Introduction

Choriocarcinoma is a rare trophoblastic malignancy characterized by poorly differentiated tumor cells, early hematogenous metastasis to multiple organs, increased β-human chorionic gonadotropin (β-hCG), and well responsiveness to chemotherapy.^[1,2]^ Choriocarcinoma is subdivided by origin and primary site into gestational and non-gestational choriocarcinoma. The latter is also called extragonadal choriocarcinoma or primary choriocarcinoma (PCC).^[3]^ PCC is highly angioinvasive with poor prognosis, and extremely rare in male patients.^[1]^ Preferentially presenting at the midline of the body,^[3,4]^ PCC primarily locates to the central nervous system, pineal body, mediastinum, retroperitoneum, lung, and liver. This phenomenon could be due to cells arising from retained primordial totipotential cells that migrate abnormally during embryogenesis.^[5]^ Primary mediastinal choriocarcinoma is more rare and found to occur predominantly in young males (Table 1).^[1–18]^ Its clinical symptoms are atypical including chest pain, cough, dyspnea, and hemoptysis (Table 1).^[3,5,6]^ Similar to other kinds of choriocarcinoma, mediastinal PCC is mainly diagnosed by increased serum levels of β-hCG, and the pathological presence of cytotrophoblast intermixed with syncytiotrophoblasts.^[6]^ However, early diagnosis is difficult because of exceeding rarity, atypical symptoms,^[3]^ overlapping differential diagnoses with other cancers, and the difficulty of obtaining a representative biopsy. The standard therapy of mediastinal PCC has not been established.^[2]^ Locoregional radiotherapy and surgery can be used for early stages together with aggressive chemotherapy, while later stages have already missed the locoregional therapy.^[19]^ The prognosis is extremely poor,^[19]^ and the majority of patients succumb within a short follow-up time (Table 1).^[1–17]^ In the case presented here, a young man was diagnosed of primary mediastinal choriocarcinoma with multiple lung and brain metastases. Six cycles of 2 different chemotherapy regimens gave a 20-week survival time from diagnosis.

**Table 1:**
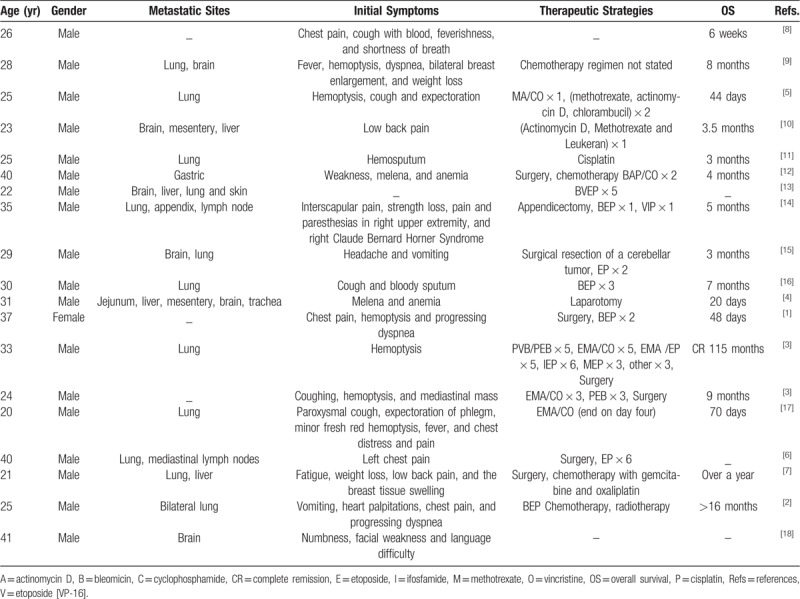
Previous reports of primary mediastinal choriocarcinoma.

## 2 Case report

**Figure 1:**
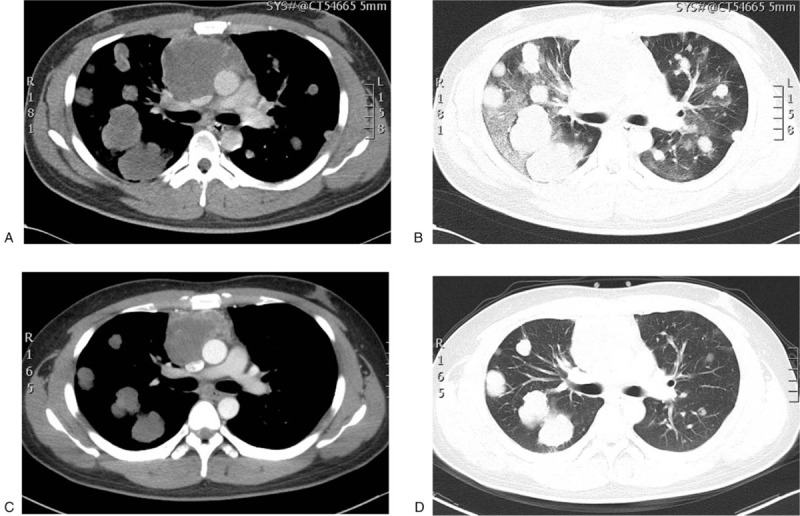
Chest CT scan shows a huge mediastinal mass and multiple bilateral pulmonary nodules prior to chemotherapy at the soft-tissue window (A) and the lung window (B). Both mediastinal and pulmonary lesions are partially reduced after four cycles of chemotherapy at the soft-tissue window (C) and the lung window (D).

A 26-year-old man presented to the Respiratory Department with a 1-month history of cough, shortness of breath, and occasional hemoptysis. His past medical, family and personal histories were unremarkable, as were physical examinations. Routine blood investigations were mostly negative, except a mild leukocytosis with predominance of granulocytes. Serum tumor markers were mostly within normal ranges, except an increase of carbohydrate antigen 125 (CA125) at 70.12 KU/l (<35.00 KU/l) and human chorionic gonadotropin (hCG) at 29.13 mIU/ml (<3.00 mIU/ml). The serum androgen level was normal, however, follicle stimulating hormone (FSH) and luteinizing hormone (LH) were decreased. Significant increases were found for estradiol (378.48 pg/ml) (11.00–44.00pg/ml), prolactin (63.74 ng/ml) (3.46–19.40ng/ml) and progesterone (1.79 ng/ml) (0.10–0.20ng/ml). Chest contrast-enhanced computed tomography (CT) revealed a large mediastinal mass and diffuse nodular opacities with blurred edges in both lungs (Fig. 1). Brain magnetic resonance imaging demonstrated multiple lesions, while CT of the abdomen, and ultrasound of the testicles and breasts revealed no abnormalities. Biopsies of a right and left lung nodule were performed, however, both failed to produce a clear diagnosis.

**Figure 2:**
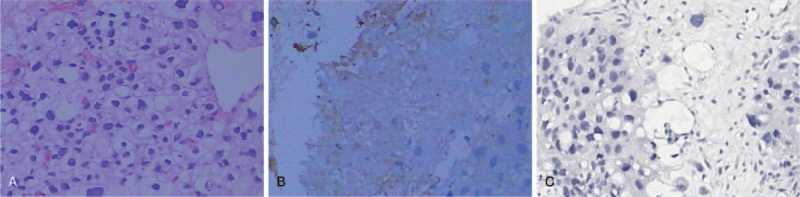
Pathological results of the anterior mediastinum mass (×200 magnification). Hematoxylin and Eosin staining (A) shows large cells close to blood vessels. Most cells have rich cytoplasm, round cell nuclei, obvious nucleolus and pathological nuclear division. Some are syncytial. Immunohistochemical stains are AFP (negative), PLAP (negative), CD30 (negative), CD117 (negative), Vimentin (negative), TTF-1 (negative), CK-Pan (positive), HCG (positive in foci) (**B**), Ki67 (60–70% positive), PD-L1 (negative) (**C**), and P40 (positive). AFP = α-fetoprotein, CD = cluster of differentiation, CK-Pan = Creatine Kinase-Pan, HCG = human chorionic gonadotropin, PD-L1 = programmed death ligands 1, PLAP = placental alkaline phosphatase, TTF = Thyroid transcription factor.

**Figure 3:**
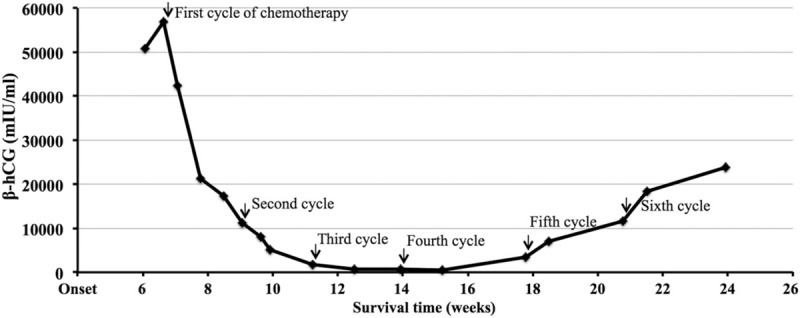
The serum β-hCG variation during treatments. Serum β-hCG decreased immediately after chemotherapy intervention, and increased again after the fourth cycle. Chemotherapy regimen change in fifth cycle did not stop it from growing.

The patient did not respond to antibiotics, hemostatics, mucolytics or bronchodilators. His condition deteriorated rapidly with frequent coughing and expectoration, recurrent hemoptysis, shortness of breath, hidrosis, chest pain and fatigue. Therefore, biopsies of both mediastinal and lung lesions were taken again, the results of which suggested choriocarcinoma (Fig. 2). A diagnosis of primary mediastinal choriocarcinoma was made based upon immunohistochemical staining of the tumor and the absence of clinical or sonographic findings of testicular involvement, according to the Multidisciplinary Team (MDT) formed by multiple departments including Oncology, Respiratory, Pathology, Radiology, Gynecology, and Urology. The patient was transferred to the Oncology Department following the diagnosis. Oncologists determined the patient's ECOG score was 3. Facial edema and engorgement of bilateral neck veins were observed, which suggested superior vena cava syndrome. Gynecomastia was noted and thought to be due to increased estradiol. The lower right lung had rhonchi without moist rales, suggesting tracheal obstruction. The patient's neck, upper chest and back were scattered with red papules in a follicular pattern, which was diagnosed by dermatologists as *Malassezia* folliculitis. Laboratory tests showed a high level of serum β-hCG (56,958.00 mIU/ml, normal range < 2.00 mIU/ml)) (Fig. 3), while urine was also β-hCG positive. Additionally, FSH and LH further decreased while CA125, estradiol, prolactin and progesterone increased. Furthermore, flow cytometry of blood lymphocytes showed a reversed CD4/CD8 ratio of 0.72 with 29% CD4^+^ T cells, though the HIV antibody tests were negative. Nonetheless, the apparent immune deficiency could be responsible for the patient's hidrosis, fatigue, *Malassezia* folliculitis and malignancy.

Because of multiple metastases, the patient was not eligible for surgical treatment or radiotherapy. Genetic testing did not find any meaningful gene mutation for existing targeted therapies, except for a missense mutation of the tumor suppressor p53 gene (TP53) at an abundance of 26.92%. Thus, systemic chemotherapy of the EMA/CO regimen (etoposide, methotrexate, actinomycin D, cyclophosphamide and vincristine) was administered. During the 4 chemotherapy cycles, the patient improved clinically in 4 areas: 1) less coughing, chest pain and hemoptysis, 2) the ECOG score lowered to 1, 3) β-hCG decreased dramatically to 442.40 mIU/ml (Fig. 3), and 4) mediastinal, pulmonary and brain lesions were partially reduced. Nonetheless, the patient subsequently deteriorated with frequent coughing, hemoptysis, sweating, fatigue, chest pain, and increased β-hCG (Fig. 3). Thus, he was given a different TP regimen (paclitaxel and cisplatin) for 2 cycles, but his illness could not be controlled. In a few days, he developed symptoms of a headache, dizziness and vomiting, which indicated intracranial hypertension caused by brain metastases. Mannitol, glycerin fructose, and dexamethasone could not stop him from getting worse. Muscle strength of the right lower extremity became 0 and a Babinski sign was present in the right foot. Intracranial hemorrhage could not be excluded. The patient and his guardian gave up on further examinations and treatments. Two weeks after discharge, he deceased with an overall survival of six and half months.

## 3 Discussion

Primary choriocarcinoma is an extremely rare, highly angioinvasive, and aggressive malignancy with poor prognosis. It occurs preferentially at the midline of the body including mediastinum.^[3]^ Primary mediastinal choriocarcinoma is exceedingly rare and presents with atypical clinical symptoms.^[1–17]^ It is mainly diagnosed by large amounts of serum β-hCG and the presence of mononuclear trophoblasts intermixed with syncytiotrophoblasts in tissues.^[19]^ However, early diagnosis is difficult due to its rare presentation, atypical symptoms, misdiagnosis as other cancers, and the difficulty of obtaining a representative biopsy due to extensive hemorrhage and necrosis,^[20]^ especially with fine-needle aspiration. Metastasis to multiple organs, like lung or brain, has usually already occurred by the time the diagnosis is made. Standardized therapeutic guidelines for mediastinal PCC have not been established. The best time for surgery or radiotherapy is inevitably missed due to early metastasis. Effective chemotherapy requires multiple chemotherapeutic agents at the same time. However, the prognosis is extremely poor,^[7,19]^ and an overwhelming majority of patients succumb within a short time, as reviewed in Table 1 showing an overall survival time from 20 days to 115 months.^[1–17]^

This case draws attention to the challenging and imperative of early detection and diagnosis of primary mediastinal choriocarcinoma. The patient wasted 1 month from the onset of initial symptoms until the first admission into the hospital. Because of the difficult diagnostic procedures, it took the patient 25 days to be finally diagnosed after three biopsies. If PCC was in the list of awareness, serum β-hCG was checked earlier, or HCG antigen was detected in the first 2 biopsies, diagnosis could have been made earlier. A sooner diagnosis allows for the rapid initiation of therapy, including the chance to resect or start radiotherapy at an earlier disease stage, which is important for better prognosis. Chemotherapy, even high-intensive regimens, could elongate the survival of PCC patients, though often the optimal curative time is missed. Therefore, primary choriocarcinoma should be recognized as a differential diagnosis of mediastinal tumors with multiple metastases, especially in young males.

Additionally, this patient showed immune dysfunction. The low levels of CD4^+^ T cells could decrease cancer immunosurveillance. Although immunotherapy is rarely administered in male mediastinal choriocarcinoma, its use may be warranted in future cases. Studies show that the programmed death receptor 1 (PD-1) and its ligand programmed death ligands 1 (PD-L1) participate in the regulation of immunosuppressive functions and the maintenance of immune tolerance.^[21]^ Thus, the PD-1/PD-L1 axis could be involved in choriocarcinoma. Both in Lu's cohort study^[22]^ and Cierna's translational study,^[23]^ PD-L1 was expressed in trophoblastic tumors, especially in gestational choriocarcinoma. However, PD-L1 expression was negative (Fig. 2) for this patient in tumor tissues and anti PD-1 therapy was not practical. Together, this suggests that primary choriocarcinoma is different from gestational choriocarcinoma in terms of PD-L1 gene expression. It is difficult to determine whether a choriocarcinoma is primary or secondary. To confirm a primary lesion in males, we must exclude ureterogenital abnormalities. However, choriocarcinoma in the genital organs may regress from a primary site before their metastatic lesions are discovered. Negative PD-L1 expression could help distinguishing extragonadal choriocarcinoma from gestational choriocarcinoma.

Furthermore, the preferred chemotherapy for gestational trophoblastic neoplasm is the EMA/CO regimen,^[3]^ which was prescribed to our patient. This patient got partial remission due to EMA/CO regimen. However, rapid drug resistance occurred after four chemotherapy cycles. The mechanism of rapid drug resistance is worthy of additional study. On the other hand, his gene tests did not lead to the ability to use a targeted therapy. Among those genes, the TP53 gene mutation could be a cause and predictor of choriocarcinoma. Whole-genome sequencing may help uncover other oncogenic factors and drug resistance mechanism, which could be targeted by alternative treatments including gene therapy. Since effective therapies are limited for primary choriocarcinoma, targeted therapy could be a hopeful therapeutic direction.

In summary, primary mediastinal choriocarcinoma is extremely rare and quite aggressive. When significantly elevated serum β-hCG levels are found in males with a mediastinal mass, choriocarcinoma should be considered as a differential diagnosis. Greater awareness and novel treatments are urgently needed to save patients with refractory mediastinal choriocarcinoma.

## 4 Conclusion

Primary male mediastinal choriocarcinoma is an aggressive malignancy with poor prognosis. Its diagnosis is obscured by atypical symptoms, overlapping differential diagnoses, and the difficulty of obtaining a representative biopsy. Earlier diagnosis and effective therapies are needed for better survival.

## Acknowledgments

We sincerely thank Edmund K. Waller, and Zachary S. Ende for reviewing the manuscript, and colleagues in Department of Oncology, Respiratory and Pathology in the Second Xiangya Hospital, Central South University for their cooperation in taking care of the patient.

## Author contributions

**Conceptualization:** Zhenhua Qiu, Yuanqiang Wu, Chunhong Hu.

**Data curation:** Yuanqiang Wu.

**Formal analysis:** Zhenhua Qiu, Yuanqiang Wu.

**Methodology:** Yuanqiang Wu, Yapeng Wang.

**Project administration:** Zhenhua Qiu, Chunhong Hu.

**Resources:** Zhenhua Qiu.

**Software:** Yapeng Wang.

**Supervision:** Zhenhua Qiu, Chunhong Hu.

**Validation:** Yapeng Wang, Chunhong Hu.

**Visualization:** Chunhong Hu.

**Writing – original draft:** Yuanqiang Wu.

**Writing – review & editing:** Yapeng Wang, Chunhong Hu.

Yuanqiang Wu orcid: 0000-0002-1731-6189.
